# Supplementary material for: Are carbon emissions trading and green financial instruments synergistic? -Comprehensive quantitative research based on content analysis
Source: PLoS One. 2024 Mar 7;19(3):e0298601. doi: 10.1371/journal.pone.0298601 (PMC10919601; doi:10.1371/journal.pone.0298601)
Supplement: S1 Data — (ZIP) [file pone.0298601.s001.zip › Data/地级市Z_F工作报告.zip!/║┌┴·╜¡/─╡╡ñ╜¡╩╨/└┤╫╘╣1⁄2╓┌║┼í╛┬φ┐╦╩2╛▌═°í┐_2019.docx]

2019年政府工作总体要求：坚持以习近平新时代中国特色社会主义思想为指导，深入贯彻落实党的十九大和中央经济工作会议精神，聚焦贯彻落实习近平总书记在深入推进东北振兴座谈会上的重要讲话和在我省考察时的重要指示精神，统筹推进“五位一体”总体布局，协调推进“四个全面”战略布局，坚持稳中求进工作总基调，坚持新发展理念，坚持推动高质量发展，坚持以供给侧结构性改革为主线，坚持深化市场化改革、扩大高水平开放，继续打好三大攻坚战，全面落实“三重三提”任务要求，按照市委“三步走”安排，统筹稳增长、促改革、调结构、惠民生、防风险、保稳定各项工作，保持经济持续健康发展和社会大局稳定，为全面建成小康社会收官打下决定性基础，奋力开创牡丹江全面振兴全方位振兴新局面。

一、2018年工作回顾

（一）强化政治担当、历史担当、责任担当，以必胜之心、打决胜之仗，三大攻坚战实现良好开局。

防范化解重大风险攻坚战首战告捷。我们持筹握算，平衡财力，保证了三大攻坚、经济发展、民生改善、社会稳定的基本支出。开展打击恶意逃废银行债务专项行动，全市金融机构不良贷款率下降0.11个百分点，存贷比提高1.5个百分点，金融业税收增长21.3%。精准脱贫攻坚战成效显著。在全省率先解决域内全部贫困人口饮水安全和危房改造问题，产业项目覆盖面、大病救治率、医疗保险参保率、“三通三有”达标率均达100%，全年15个村脱贫出列、3174人脱贫退出，宁安、爱民、西安、东安贫困人口全部脱贫，全市贫困发生率下降到0.18%，成效居全省前列。污染防治攻坚战深入推进。开展“清零”行动，彻底取缔城区10蒸吨以下燃煤小锅炉，秸秆综合利用率、离田率分别达到42%和74.6%，市区空气优良天数增加12天。河湖长制由全面建立转向初见成效，开展镜泊湖污染治理百日攻坚，拉开湖区污染治理序幕。实施农业“三减”、整治违法违规“大棚房”、治理水土流失，黑土地保护专项行动全面展开，餐厨废弃物处置项目近期投入运行，亿丰煤气等高污染企业和产能15万吨以下小煤矿全部关停。主要污染物减排指标达到省定标准。

（二）向经济建设这个中心聚焦发力，扎实推进“三大攻坚任务”，高质量发展取得新成效。

经济运行总体平稳。开展规上企业保卫行动，落实24条稳增长促发展政策措施，停产规上工业企业从81户减少到28户，规上工业增加值由负转正，全年预计增长3.5%，预计地区生产总值由持平实现增长，城乡居民人均可支配收入分别增长7%和8%。产业项目建设取得新进展。重点推进“老字号”千万元以上技改项目26个、“原字号”“新字号”亿元以上项目78个和85个；新开工万泰木业等亿元以上产业项目100个，实施精准招商，引进亿元以上项目68个，争取政策性资金182亿元。现代农业实现新提升。深化农业供给侧结构性改革，加快发展特色高效精品农业，食用菌栽培规模增长10%，蔬菜基地和林下经济面积均增长9%，龙稷马铃薯、谢记南瓜深加工等8个亿元以上“两头两尾”项目投产运营。消费升级拓展新空间。加快发展“旅游+”产业，开复工牧心冰酒小镇等“旅游+”项目13个，牡丹电子体验馆等3个亿元以上项目竣工，成功举办国际马拉松赛、镜泊湖冬捕节。加快发展商贸物流新业态，总投资80亿元的月星环球港开工建设，电子商务企业园入驻企业60户，全市电商交易额增长27%、居全省第二位；全市新文创企业发展到40户，全市文化产业增加值突破50亿元。创新驱动凝聚新动能。科技企业孵化器、众创空间分别达到43家和40家、在孵企业357户，北一半导体模块填补省内空白，成功承办2018中国核产业发展高峰论坛。设立创业专项资金，扶持创业1995人、带动就业10073人；成立引进人才服务中心，设立人才专项资金，首批引进各领域急需紧缺人才71人，培养高技能人才3390人，培训企业家350人。对外开放迈出新步伐。牡丹江机场迁建选址获批，哈牡客专通车，牡绥快铁提速，我市走进“高铁时代”。深化改革和优化营商环境取得新成效。稳妥推进政府机构改革，有序完成机构组建挂牌和人员转隶工作；新注册企业审批时限减至30分钟，全市新增市场主体2.7万个、网上注册企业增长81.9%。与浪潮集团开展战略合作，全面启动“数字政府”建设。侯卫东官场笔记

（三）坚持以人民为中心的发展思想，持续提高民生投入比重，全力以赴办实事、补短板、惠民生。

30件利民实事基本完成，民生支出比重保持在80%以上。就业社保工作扎实推进。新增城镇就业5.95万人，五项社保新增参保13.9万人，机关事业单位和企业退休人员养老保险标准分别提高3.9%和5.3%，市区失业金标准提高32%。人居环境持续改善。新火车站和智能化城管平台投入使用，11条主次干道建成通车，新安街焕然一新，市区新增停车泊位7042个、绿地45万平方米。社会事业稳步发展。义务教育学校标准化建设全部完成，第八中学新校舍投入使用，立新小学等江南新校区建设收尾，超大班额全部消除；持续推进“健康牡丹江”行动，基层医疗卫生机构覆盖率保持100%；新建全民健身路径50处，公共文化云平台投入运行。社会治理格局日益完善。全市安全生产事故起数下降6.25%，全面开展扫黑除恶专项斗争，侦办黑恶势力犯罪案件26起、治安案件分别下降1.8%和9%。全省房地产领域信访问题专项整治积案全部办结，8354户逾期未回迁居民回迁，9472户居民房产办证难问题得到解决。

（四）切实加强政府自身建设，努力提高施政能力和服务水平，以勤政廉政服务全市人民。

全面落实政治生态和意识形态工作责任制，制定市政府党组“工作规则”“议事规则”，扎实推进“两学一做”学习教育常态化制度化，组织开展解放思想推动高质量发展大讨论。深入开展“法治建设年”活动，严格落实会前学法制度。自觉接受人大、政协和社会监督，办理人大代表议案建议268件、政协委员提案251件，办复率均为100%。认真落实党风廉政建设“一岗双责”，严格执行厉行节约相关规定，“三公”经费支出只减不增。统计、老龄、气象、地震、档案、人防、边防、民兵预备役、妇女儿童、残疾人、红十字等工作都取得新成绩，驻牡中省直部门、驻牡部队为地方发展做出突出贡献。

二、2019年重点工作任务

全市经济社会发展预期目标：地区生产总值增长5%以上，固定资产投资增长3.5%，一般公共预算收入增长3%左右，规上工业增加值增长5%，社会消费品零售总额增长5%左右，进出口总额增长5%，城乡居民人均可支配收入均增长7%，万元GDP综合能耗下降3.2%。

（一）坚持发展第一要务不动摇，突出重点稳增长、强后劲。

一是稳住工业这个全市经济的“定盘星”。深入实施规上企业培育专项行动，全年新增规上企业30户、税收增长10%以上，工业投资增长15%以上。二是扭住产业项目这个振兴发展的“牛鼻子”。开辟重点项目审批“绿色通道”，全市新开工亿元以上项目100个，霖润天然纤维素等30个亿元以上项目竣工投产。三是抓住招商引资这个贯穿经济的“生命线”。用好对口合作机制，鼓励引导东莞企业在牡设立出口加工区、制造业配套园区、家具产业园区和科技孵化园区；全年引进亿元以上项目100个，引进域外资金和实际利用外资增幅高于全省平均水平。四是做好结构调整这篇高质量发展的“大文章”。重点推进首控50万吨沥青、恒丰纸业食品包装纸、牡丹江烟厂等29个亿元以上技改升级项目；加快建设恒源食品、汉麻全产业链、镈浩石墨等精深加工项目，推进穆棱玄武岩连续拉丝等“新字号”项目。五是壮大民营经济这支极具活力的“生力军”。实施“重点民营企业五年倍增计划”，加大融资服务力度，推进重点企业上市进程，掀起新一轮民营经济发展热潮。

（二）以创新激发内生动力，重聚产业发展新动能。

一是适应消费升级需求发展新产业。实施旅游产业转型升级三年行动计划，加快镜泊湖智慧旅游景区、狼图腾旅游综合体、莲花休闲度假中心等“旅游+”项目建设；围绕“哈亚镜雪牡”整合旅游资源，加快开发冰雪、跨境、休闲度假、养生养老等旅游产品，加大旅游市场综合整治力度；深化与当当、腾讯、阅文集团合作，谋划建设新文创个性化总部基地，加快建设保利新文化创意小镇、爱熊猫小镇等项目，支持国品、华创新文创等文化产业园建设。二是运用新技术增创新优势。实施“高新技术企业培育”行动，达标科技型企业和国家级高新技术企业分别达到80户和35户；加快北方合金先进精密刀具研发中心建设；利用牡丹江浪潮数字经济平台和恒丰恒舢工业互联网平台，引导具备条件企业建设数字化车间、智能化工厂，提高工业信息化水平。三是以新模式新业态拓展新空间。加快推进月星环球港、新城吾悦广场、中农批（牡丹江）东北亚物流产业园、市经开区及绥芬河电商产业园建设，发展壮大本地电商平台，全市电子商务交易额增长10%。深化与浪潮集团战略合作，加快数字城市建设。四是强化双创平台和人才支撑。实施“十百千”主体培育计划，实现科技企业孵化器、众创空间“双50”目标、在孵企业达到500户以上。全面落实市委“1+N”人才政策，全力留住现有人才、引进紧缺人才，为振兴发展提供人才保障。

（三）坚持农业农村优先发展，推动乡村全面振兴。

一是加快发展现代农业。新增棚室菌2000万袋、设施菜2000亩、林下经济10万亩，畜牧业产值增长5%。加快一二三产融合进度，推进祥源米业、纳豆生物、中林集团鹅屠宰加工等亿元以上项目10个，全市新增农业产业化龙头企业10户；加大绿色生态农产品品牌培育力度，80%以上绿色有机食品认证企业进入省农产品质量安全追溯公共服务平台；尽最大努力防控以非洲猪瘟为重点的重大动物疫病发生。二是提升农村生活品质。新建农村公路300公里，农村居民饮水安全梯次推进，完成省定农危房改造任务，全市500户以上的行政村实现交邮合作全覆盖。深入推进“农村人居环境整治三年行动”，扎实推进“厕所革命”。三是扎实推进新一轮农村改革。深化农村土地制度改革，推广土地承包经营权抵押贷款、入股产业化经营等模式，推进农信社产权制度改革和供销合作社综合改革，落实国有林场改革政策，加强“一懂两爱”农村工作队伍建设，推进新型经营主体建设，农民专业合作社规范社达到270个。

（四）统筹推进重点领域改革，重塑投资营商新环境。

一是持续深化“放管服”改革。实行“多证合一”“多审合一”“多图联审”，逐步实现投资服务“一扇门”、审批受理“一扇窗”、共享数据“一网办”，“最多跑一次”审批事项占比提高10个百分点以上，审批时限、要件、环节减少20%；探索实施企业简易注销登记改革，加快“数字政府”和民生服务大厅建设，推进“四零”承诺服务创建向基层延伸。二是扎实推进政府机构、事业单位和国资国企改革。高标准完成市政府各部门“三定”，加快推进内设机构大科（室）制改革；有序推进事业单位改革，事业编制和事业机构各精简10%；深化国资国企改革，积极推进镜泊湖管理体制和新闻传媒集团改革。三是持续优化营商环境。深入开展整顿机关作风优化营商环境活动，建立营商环境评价体系，健全“五位一体”举报受理平台，严肃查处破坏营商环境案件；加快“诚信牡丹江”建设，推进失信违诺专项清理，完善政府守信践诺机制。

（五）深度融入共建“一带一路”，建设开放合作新高地。

一是加快绥东重点开发开放试验区等重大开放合作平台建设。加快试验区相对集中行政许可权改革和“五个中心”建设，强化绥芬河综合保税区承载功能，量身定制比市经开区更优惠的保税物流中心（B型）招商政策，做好绥波跨境经济合作区前期工作。二是加快集疏运体系建设。重点加快建设绥芬河国际集疏运物流平台，推进东宁口岸升级国际客货运输口岸，加快绥东机场、东珲公路、牡佳客专建设，同步谋划推动牡符高铁、绥格铁路、牡敦客专和东北亚物流信息港建设；支持华晟内陆港多式联运示范工程建设，确保“哈绥符釜”班列持续运营。三是促进贸工一体化外向型产业发展。重点抓好食品加工、林木林纸、轻工建材等进出口加工基地建设，加快建设进出口加工产业集聚区和跨境连锁加工区。四是扩大对外交流合作。积极参与中俄地方合作交流年、中俄博览会等活动，办好中俄体育交流大会、中国国际口岸贸易博览会，推动对外合作在科技教育、医疗康养、文化体育等领域不断深化。

（六）科学统筹精准施策，重构协调发展新格局。

一是提升中心城市建设管理水平。扎实推进全国文明城市创建工作，加快建设江南新城区，建设公铁联运中心，实现公铁“零换乘”，加快构建“快进慢游”综合交通网络；推进保障性住房建设，改造市区棚户区500户；天然气入户新增1万户，推进垃圾焚烧发电和城郊垃圾收运一体化项目建设。二是促进城乡区域一体化发展。建立“城市体检”评估机制，改革创新开发区体制机制，推动市经开区剥离社会职能，回归经济职能；科学界定市区事权，加强市区两级职责衔接、工作联动；推动县域经济与城区经济差异化发展，确保县域经济分类考核重点指标、重点工作保位争先。三是创新区域协调发展机制。高标准编制国土空间总体规划，统筹衔接土地利用、城市风貌、城乡建设、产业布局、旅游发展等规划；研究推动绥东重点开发开放试验区政策向牡丹江全域辐射、与俄超前发展区和自由港政策对接，鼓励县（市）区项目在各园区间“飞地建设”；认真研究、抢先布局我市在省会“两小时经济圈”的功能定位，加快融入哈长城市群，实现协同发展。

（七）加强生态文明建设，巩固提升绿色发展优势。

一是继续打好污染防治攻坚战。高标准完成中央环保督察“回头看”整改任务，有效解决秸秆露天焚烧和综合利用，城市空气质量优良天数比例达到90%以上，县级城市建成区内燃煤小锅炉淘汰率达到80%；扎实推进河湖长制，对全市14条重要河流设置55个县（市）界、镇（乡）界水质监测断面，饮用水水源地水质达标率100%；实施黑土地战略性保护，确保黑土地不减少、不退化。二是加大生态保护和修复力度。加快国家生态文明先行示范区试点建设，开工建设林海水库，加快荒沟电站建设，逐步恢复东北虎豹国家公园生态功能；完成基本草原清查工作，推动矿山、裸露山体、坡地综合治理，治理水土流失28万亩、植树造林8万亩。三是推动形成绿色发展方式和生活方式。推进中俄清洁能源产业园、聚生湶环保处理装备制造等项目建设，支持长青环保、瑞禾农业等企业上规模、拓市场、增效益；借力北京冬奥会，推进寒地冰雪经济快速发展。

（八）更加关注补齐民生领域短板，切实提升民生保障水平。

高质量办好10件惠民实事，让群众共享振兴发展成果。一是坚决打好打赢精准脱贫攻坚战。全力解决因病因残致贫等最难解决的问题，实现现行标准下建档立卡988户2057人贫困人口全部脱贫、贫困村全部出列。二是着力抓好就业社保。启动“智慧人力资源市场”建设，新增城镇就业5.1万人以上，城镇登记失业率控制在4.3%以内。提高城乡居民基础养老金标准，确保城镇退休人员养老金及时足额发放。提高城乡低保和特困供养保障标准，大力发展居家社区养老服务，逐步实现“一刻钟社区服务圈”全覆盖。三是统筹发展社会事业。提高高中阶段教育普及水平，推动学前教育规范科学发展，推进二十一中异地建设，平稳启用立新小学等江南新校区。推进“健康牡丹江”建设，有序推进分级诊疗工作，建设牡丹江健康信息惠民服务平台。实施文化惠民工程，编制全市红色文物名录。四是加强和创新社会治理。强化四城区住宅物业管理主体责任，完成城市社区换届选举工作，依法管理民族宗教事务，守牢生产、食品、药品、特种设备安全底线，扎实推进“六大工程”建设，持续深化扫黑除恶专项斗争，建设更高水平的“平安牡丹江”。

（九）全面加强政府自身建设，切实提升政府治理能力和水平。

树牢“四个意识”，坚定“四个自信”，坚决做到“两个维护”，精心组织“不忘初心、牢记使命”主题教育，不折不扣贯彻落实好上级和市委各项决策部署。深入开展解放思想推动高质量发展大讨论，大兴学习和调查研究之风，不断提高解决问题、推动发展的能力。扎实推进法治政府建设，严格执行政府工作规则和“三重一大”制度，加强和创新“双随机一公开”等监管方式，整合组建市场监管、生态环境保护、交通运输等综合执法队伍。自觉接受人大法律监督、政协民主监督以及社会各界监督，高质量办好人大议案、建议和政协提案。加强政府内部管理，继续压缩一般性支出，切实提高财政资金使用效益。严格落实全面从严治党主体责任和“一岗双责”，坚决执行中央八项规定和省委、市委改进工作作风密切联系群众规定，努力建设让党放心、让人民满意的服务型政府。
